# Determination of Seed Soundness in Conifers *Cryptomeria japonica* and *Chamaecyparis obtusa* Using Narrow-Multiband Spectral Imaging in the Short-Wavelength Infrared Range

**DOI:** 10.1371/journal.pone.0128358

**Published:** 2015-06-17

**Authors:** Osamu Matsuda, Masashi Hara, Hiroyuki Tobita, Kenichi Yazaki, Toshinori Nakagawa, Kuniyoshi Shimizu, Akira Uemura, Hajime Utsugi

**Affiliations:** 1 Department of Biology, Faculty of Sciences, Kyushu University, Fukuoka, Japan; 2 Tsukuba Research Institute, Sumitomo Forestry Co., Ltd., Tsukuba, Ibaraki, Japan; 3 Forestry and Forest Products Research Institute, Tsukuba, Ibaraki, Japan; 4 Department of Agro-environmental Sciences, Faculty of Agriculture, Kyushu University, Fukuoka, Japan; 5 Hokkaido Research Center, Forestry and Forest Products Research Institute, Sapporo, Hokkaido, Japan; Murdoch University, AUSTRALIA

## Abstract

Regeneration of planted forests of *Cryptomeria japonica* (sugi) and *Chamaecyparis obtuse* (hinoki) is the pressing importance to the forest administration in Japan. Low seed germination rate of these species, however, has hampered low-cost production of their seedlings for reforestation. The primary cause of the low germinability has been attributed to highly frequent formation of anatomically unsound seeds, which are indistinguishable from sound germinable seeds by visible observation and other common criteria such as size and weight. To establish a method for sound seed selection in these species, hyperspectral imaging technique was used to identify a wavelength range where reflectance spectra differ clearly between sound and unsound seeds. In sound seeds of both species, reflectance in a narrow waveband centered at 1,730 nm, corresponding to a lipid absorption band in the short-wavelength infrared (SWIR) range, was greatly depressed relative to that in adjacent wavebands on either side. Such depression was absent or less prominent in unsound seeds. Based on these observations, a reflectance index SQI, abbreviated for seed quality index, was formulated using reflectance at three narrow SWIR wavebands so that it represents the extent of the depression. SQI calculated from seed area-averaged reflectance spectra and spatial distribution patterns of pixelwise SQI within each seed area were both proven as reliable criteria for sound seed selection. Enrichment of sound seeds was accompanied by an increase in germination rate of the seed lot. Thus, the methods described are readily applicable toward low-cost seedling production in combination with single seed sowing technology.

## Introduction

*Cryptomeria japonica* (Linn. fil.) D. Don. (sugi) and *Chamaecyparis obtusa* Sieb. et Zucc. (hinoki) are coniferous trees in the Cupressaceae family and are two of the major plantation species in Japan [1]. Two thirds of Japan's total land area are covered with forests, of which as much as 30% is accounted for by sugi and hinoki plantations. This is due on one hand to their value as building materials, and on the other hand to large-scale afforestation activities in the post-World War II period [2, 3]. Hence, wide expanse of sugi and hinoki forests have reached their rotation age. In the meantime, however, Japan started to depend on imports to meet the demand for timbers, and this diminished the competitiveness of local forest products and made domestic forestry unprofitable. As a result, many of mature forest plantations have become neglected without proper management. To make matters worse, over-abundance of such monospecific plantations has brought about another serious problem; airborne pollen released from adult sugi and hinoki trees is causing hay fever symptoms in more than one third of all citizens [3]. Thus, thinning to prolong the rotation age or clear-cutting of overaged plantations is a pressing issue to the forest administration in Japan [2].

Whichever option was adopted, it is important to premeditate how current plantations should be replanted after the final cut, taking multifaceted role of forests into consideration. Cessation of plantation after clear-cutting could increase the risk of landslides and debris flows [4], and also cancel the CO_2_ fixation effect of the forests, leading to a net increase in CO_2_ emissions [5]. However, high labor cost for reforestation has hampered the promotion of planted forest regeneration [2, 6].

One of the potential solutions that can reduce the total cost and labor for replanting is the use of container seedlings. They are grown in small tubular containers and do not need to be detached from nursery bed before planting, which allows to keep root systems intact. Because of this, container seedlings can outperform traditional bare-root seedlings in growth, survival and establishment [7]. Moreover, standardized size and shape of the containers are promising for large-scale, year-round, and semi-automated production of reforestation seedlings.

Setting aside a concern about whether, how, and to what extent planted forests should be transformed into semi-natural forests [2], one of the factors that have delayed the prevalence of container seedlings of sugi and hinoki is extremely variable and usually low germination rate of their seeds [8]. With low-germination seed lots, single seed sowing can give rise to missing plant containers due to failure of germination, while thick sowing will call for the labor of thinning out. The primary cause of the low germinability has been attributed to highly frequent formation of anatomically unsound seeds [9]. Among forestry workers and researchers in Japan, one of them is known as “shiina” and another as “shibudane”, which are characterized by empty and blackish-brown tannin-like contents, respectively. Much to our inconvenience, however, most of these unsound seeds cannot be distinguished from sound germinable seeds by visual observation with naked eyes and other common measuring methods.

To enable low-cost production of container seedlings for sugi and hinoki forest regeneration, hyperspectral imaging technique was used to identify multispectral wavebands optimal for separating sound seeds out of unsorted seed lots. Here we demonstrate how reflectance within SWIR wavebands can be used to evaluate seed soundness and thereby contribute to improve the germination rate of sugi and hinoki seed lots.

## Materials and Methods

### Seed materials

Seeds of sugi and hinoki were obtained from Research Planning and Coordination Department of The Forestry and Forest Products Research Institute (FFPRI; Ibaraki, Japan). In this department, seeds from specified mother trees in various parts of Japan are gathered for distribution to academic and industrial researchers, and hence the germination rate of each seed lot, which is available on request, is routinely examined based on the methods prescribed by International Seed Testing Association (ISTA) [10]. After harvesting, seeds are divided into small groups and stored at -5°C in sealed evacuated cans until distributed. Experiments were conducted immediately after receiving the seeds or, if necessary, they were stored in desiccator at 4°C. Information on all seed lots used in this study is summarized in Table 1.

**Table 1:** *Cryptomeria japonica* and *Chamaecyparis obtusa* seed lots used in this study.

Species		Germination Rate			Seed Group			
						Unsound		
	Seed Lot^*a*^	Initial (Highest)	Date Tested	This Study (Aug. 2014)	Sound	Shibudane	Others	*N* ^*b*^
***Cryptomeria japonica* (Sugi)**	Ibaraki SA1	41%	Apr. 1996	-	37%	40%	23%	100
	Ibaraki SA2	58%	Jul. 2006	-	76%	23%	1%	100
	Ibaraki SA3	62%	May 2012	43.9%	66.7%	32.8%	0.5%	180
	Ibaraki SA4	47%	Jun. 2012	-	41%	44%	15%	100
	Ibaraki SC1	37%	Dec. 1998	-	51%	33%	16%	100
	Ibaraki SD	24%	Dec. 2002	-	58%	22%	20%	100
	Kumamoto SG	13%	May 1996	-	13%	74%	13%	100
	Kumamoto SH	8%	Oct. 1997	-	9%	85%	6%	100
	Nagano SI	28%	Aug. 2000	-	32%	59%	9%	100
	Shimane SJ	18.3%	Feb. 2014	14.4%	17.2%	50.0%	32.8%	180
***Chamaecyparis obtusa* (Hinoki)**	Ibaraki HB1	41%	Jun. 2012	17.2%	32.8%	48.3%	18.9%	180
	Ibaraki HB2	77%	Jun. 2012	-	72%	24%	4%	100
	Ibaraki HB3	69%	Jul. 2012	-	69%	23%	8%	100
	Ibaraki HC2	9%	Dec. 1998	-	7%	85%	8%	100
	Ibaraki HE	47%	May 1996	-	54%	39%	7%	100
	Ibaraki HF	25%	May 2003	-	31%	67%	2%	100
	Gifu HK	32%	May 1999	-	46%	50%	4%	100
	Kochi HL	58%	Jun. 1996	-	55%	39%	6%	100
	Kochi HM	53%	Jun. 2009	21.1%	58.9%	34.4%	6.7%	180
	Kyoto HN	19%	Apr. 2004	-	17%	79%	4%	100

^*a*^ The seed lot name includes native prefecture followed by codes for species (S, sugi; H, hinoki) and the seed orchard, respectively, in a single capital letter. If necessary, the name is suffixed by a reference number of the mother tree in each seed orchard.

^*b*^ Number of seeds used to determine germination rate (this study) and proportions of each seed group.

### Microscopic observation of seed structure

Seeds were embedded in Tissue-Tek optimal cutting temperature (O.C.T.) compound (Sakura Finetek Co., Ltd., Tokyo, Japan) and then cooled before mounting on the specimen holder. The transverse face of each seed was cut with a cryostat microtome (HM 505 E; MICROM International GmbH, Walldorf, Germany), and the cutting plane was photographed using an SMZ1000 stereomicroscope equipped with a DXM-1200C digital camera (Nikon; Tokyo, Japan).

### Hyperspectral reflectance imaging

The configuration of hyperspectral imaging apparatus is basically identical to the one reported previously [11]. Two line-scanning hyperspectral cameras, one is sensitive to visible (VIS) to near-infrared (NIR) wavelengths (400–980 nm; model VNIR-200R) and the other to NIR to SWIR (1,250–2,500 nm; model SWIR-200R), were purchased from Themis Vision Systems LLC (VA, USA) and Emerging Technologies LLC (WI, USA), respectively. The successor products of these are now available from PhiLumina LLC (MS, USA). VNIR-200R is equipped with a silicon charge-coupled-device (CCD) detector and is capable of acquiring 1,392 × 1,000 pixel images with 12-bit digitization and at 1.3 nm spectral resolution (with 12 μm-wide entrance slit). SWIR-200R is equipped with a cooled photoconductive mercury-cadmium-telluride (MCT) detector and is capable of acquiring 14-bit 320 × 200 pixel images at 7.7 nm spectral resolution (with 25 μm-wide slit). With our experimental setups, the resulting spatial resolution of images produced by the VNIR and SWIR cameras were ca. 156 (area of 223 mm × 165 mm) and 106 (80 mm × 46 mm) pixel per inch (ppi), respectively. As the epi-illumination light source, two double-ended halogen lamps were installed in the imaging apparatus. Tubular bulbs with 250W and 100W outputs were used for imaging with the VNIR and SWIR cameras, respectively. A full-area hyperspectral image of a 50% grey Spectralon diffuse reflectance target (SRT-50-100; Labsphere Inc., NH, USA) was used as the reference to transform raw radiance images from the SWIR camera to reflectance images by a pixelwise linear interpolation (calibration). For calibration of VIS/NIR radiance images, a sheet of white polytetrafluoroethylene (PTFE) filter paper (PF100; ADVANTEC Toyo Kaisha Ltd., Tokyo, Japan), in place of a Spectralon target, was used as the 90% reflectance standard. The algorithms for hyperspectral image calibration are implemented in HyperVisual image capturing software provided by the camera manufacturers. Prior to hyperspectral imaging, seeds were aligned on a matt black-finished polyvinylchloride (PVC) plate, on which 18 flat-bottomed wells with 1 cm diameter and 1 mm depth were laid out in a 6 × 3 grid format.

### Seed germination test

After hyperspectral imaging, the seeds on PVC plates were transferred to a flatbed scanner (ES-H7200; Seiko Epson Corp., Nagano, Japan) placed upside down and scanned in red-green-blue (RGB) format without any color correction. Out of 20 plates that have passed through hyperspectral and RGB imaging, 180 seeds on half of the plates were subjected to a cutting test, while the rest to a germination test. Seed lots not processed for the imaging procedures (100 seeds for each lot) were subjected directly to a cutting test (Table 1). The details of cutting test is described in the results. Germination test was carried out according to the methods prescribed by ISTA [10]. In brief, seeds of both sugi and hinoki were placed on a sheet of moist filter paper layered on 0.9% (w/v) agar plate and incubated for 28 days test period at an alternating light and temperature condition of 8 h light at 30°C and 16 h darkness at 20°C. No pretreatment for germination was applied in this study.

### Seed viability test

Viability of sound seeds in seed lots subjected to both cutting and germination tests were evaluated by tetrazolium staining [10]. Potential sound seeds, as presumed by the single and dual parameter methods described in the results, were imbibed on a sheet of moist filter paper for 24 h under continuous light at 22°C, and then bisected with a razor blade in a longitudinal direction. For each seed lot, the first 24 seeds that were filled with white endosperm were transferred individually to microtiter plate wells dispensed with 0.1% (w/v) solution of 2,3,5-triphenyltetrazolium chloride (Nacalai Tesque, Inc., Kyoto, Japan). The plate was sealed and incubated for 3 h at 40°C. Seeds containing a red-stained embryo were considered as viable.

### Gas chromatography-mass spectrometry analysis

For extraction of lipophilic compounds, 50 mg of sugi and hinoki seeds were immersed in 1.5 mL of diethyl ether for 24 h with occasional agitation at room temperature. Gas chromatography-mass spectrometry (GC-MS) analysis was performed with a model 5975B inert XL MSD system (Agilent Technologies Inc., CA, USA) equipped with an HP-5ms capillary column (30 m × 0.25 mm i.d., 0.25 mm film thickness; Agilent Technologies Inc.). The inlet temperature was held at 250°C, while the column temperature was programmed at 40°C for 3 min, increased to 250°C at 3°C min^-1^, then to 300°C at 10°C min^-1^, and finally maintained at this temperature for 5 min. The helium carrier gas was set at a flow rate of 1 mL min^-1^. One microliter of the extract was injected in splitless mode. Mass spectrometer was operated in total ion current (TIC) mode at 70 eV of ionization energy with a scan range from 35 to 550 amu. Identification of individual compounds was based on comparison of their retention indices and mass fragmentation patterns with those on the Wiley Registry 9th/National Institute of Standards and Technology (NIST) 2008 mass spectral library [12].

### Software development and data analysis

**Fig 1:**
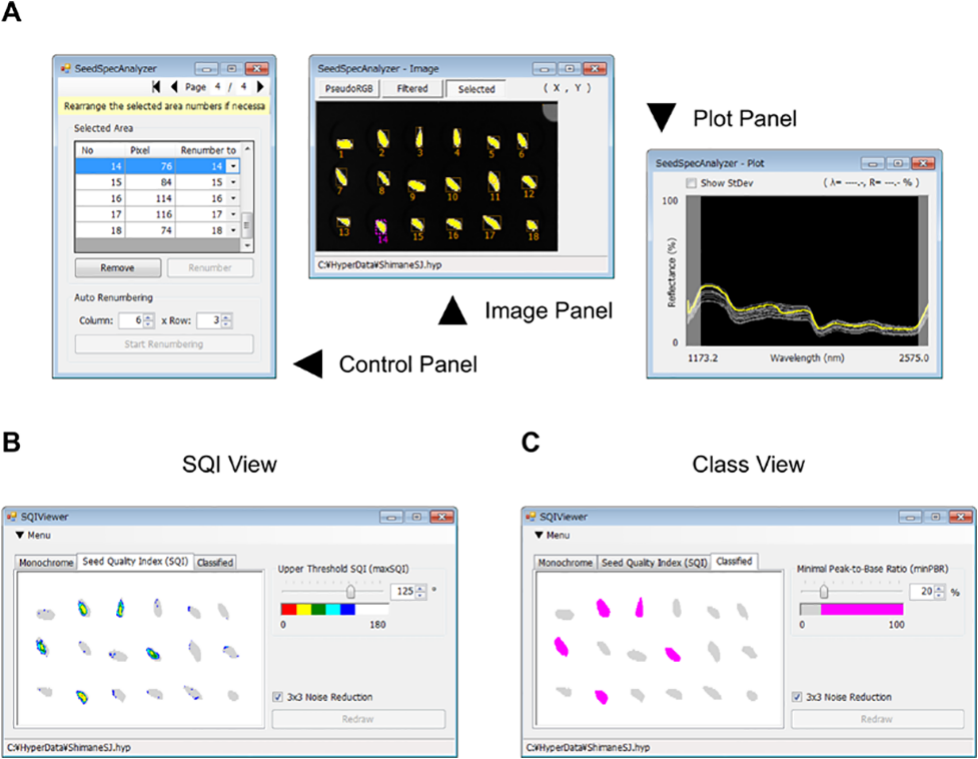
Hyperspectral image analysis software developed for this study.

The software shown in Fig 1, which is provided as S1 File (S1 Text for methods of operation), was developed in managed C# code using Microsoft Visual Studio 2010 Professional with extensions of Emgu CV (version 2.3.0.1416; http://www.emgu.com/), a. NET wrapper to the OpenCV image processing library (version 2.3.1: http://www.opencv.org/), and Math.NET Numerics (version 3.1.0, http://numeric.mathdotnet.com/), which provides methods and algorithms for scientific computations including matrix and statistics-related functions. The software works on 64-bit versions of Microsoft Windows operating systems with. NET Framework 4 runtime installed. For detailed analysis of the statistics related to the procedures of sound seed selection, reflectance or SQI data were exported as tab-separated-values (TSV) format files from the above software and reloaded in Microsoft Excel or R statistical software (version 3.1.1, http://www.r-project.org/). Three dimensional graphs and heat maps were drawn using RINEARN Graph 3D software (version. 5.5.34, http://www.rinearn.com/graph3d/).

## Results

### Correlation between germinability and anatomical soundness of sugi and hinoki seeds

**Fig 2:**
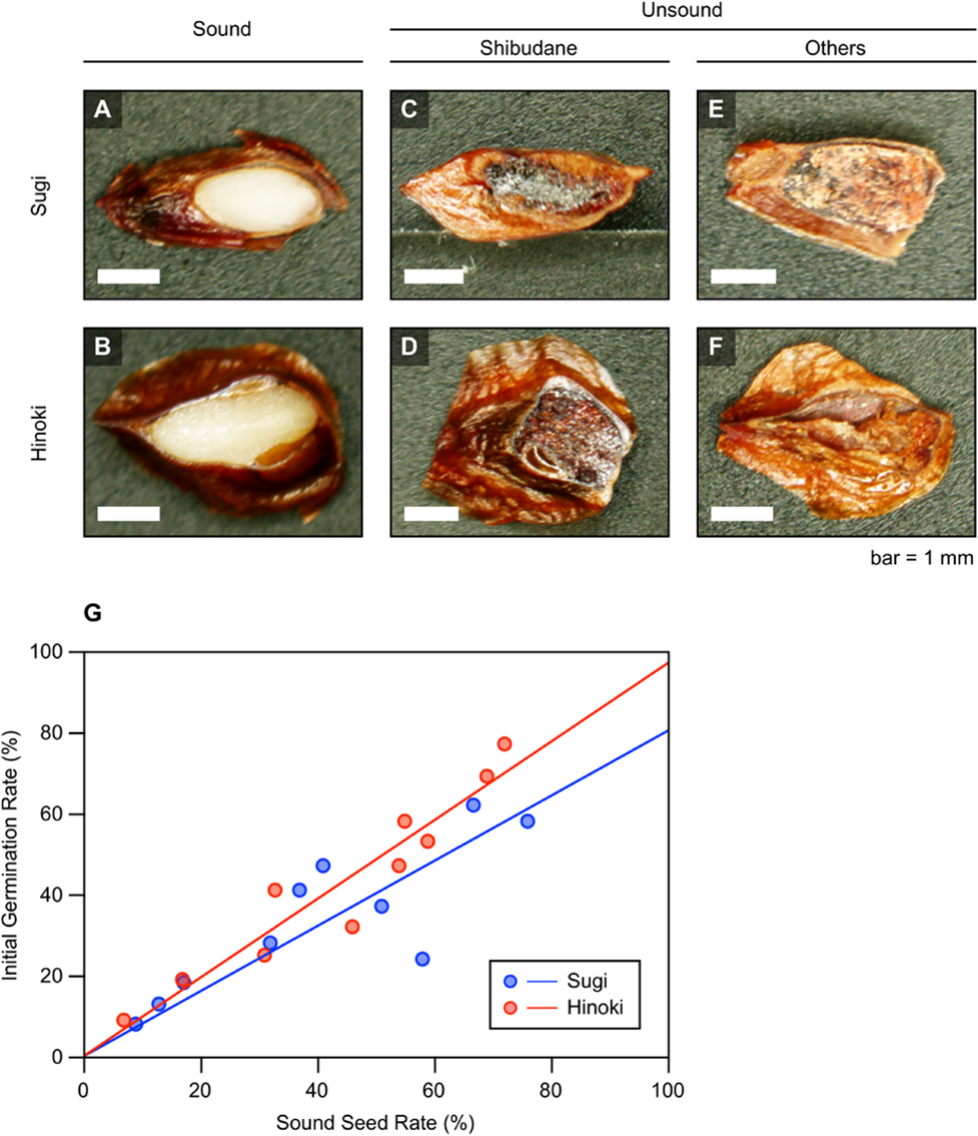
Anatomical features of sound and unsound seeds of *Cryptomeria japonica* (sugi) and *Chamaecyparis obtusa* (hinoki).

To confirm whether low natural germination rate of sugi and hinoki seeds is due to highly frequent formation of anatomically unsound seeds, 10 independent seed lots for each tree species were obtained (Table 1). According to the records of routine germination test carried out by the seed provider, the initial germination rate of each seed lot, in which the impact of quality deterioration during storage should be minimal, was highly variable, with values ranging from 8% to 62% for sugi and 9% to 77% for hinoki (Table 1 and Fig 2G). All the obtained seed lots were subjected to a cutting test, which was carried out by bisecting a total of 100 or 180 seeds for each lot using a small pair of scissors. Based on the cross sectional color and appearance, they were classified into three groups. In first step, seeds filled with white waxy endosperm (Fig 2A and 2B) were classified as “sound” and the others as “unsound”. Unsound seeds were further classified into two additional groups; one as “shibudane” (Fig 2C and 2D) and the rest as “others” (Fig 2E and 2F). The group “others” included empty seeds, whereas the majority was characterized by spongy or ligneous substance that could be an underdeveloped endosperm. The results of cutting test are also summarized in Table 1.

Because anatomical soundness was considered a prerequisite for seeds to germinate, correlation between the proportion of sound seeds and the initial germination rate was examined for both of the sugi and hinoki seed lots on a scatter plot. As shown in Fig 2G, the 10 data points for each tree species were mostly aligned along an upsloping regression line, which was close to the identical line. This suggested that enrichment of sound seeds is one of promising approaches to produce sugi and hinoki seed lots with a high germination rate.

### General features of sound and unsound sugi and hinoki seeds

**Fig 3:**
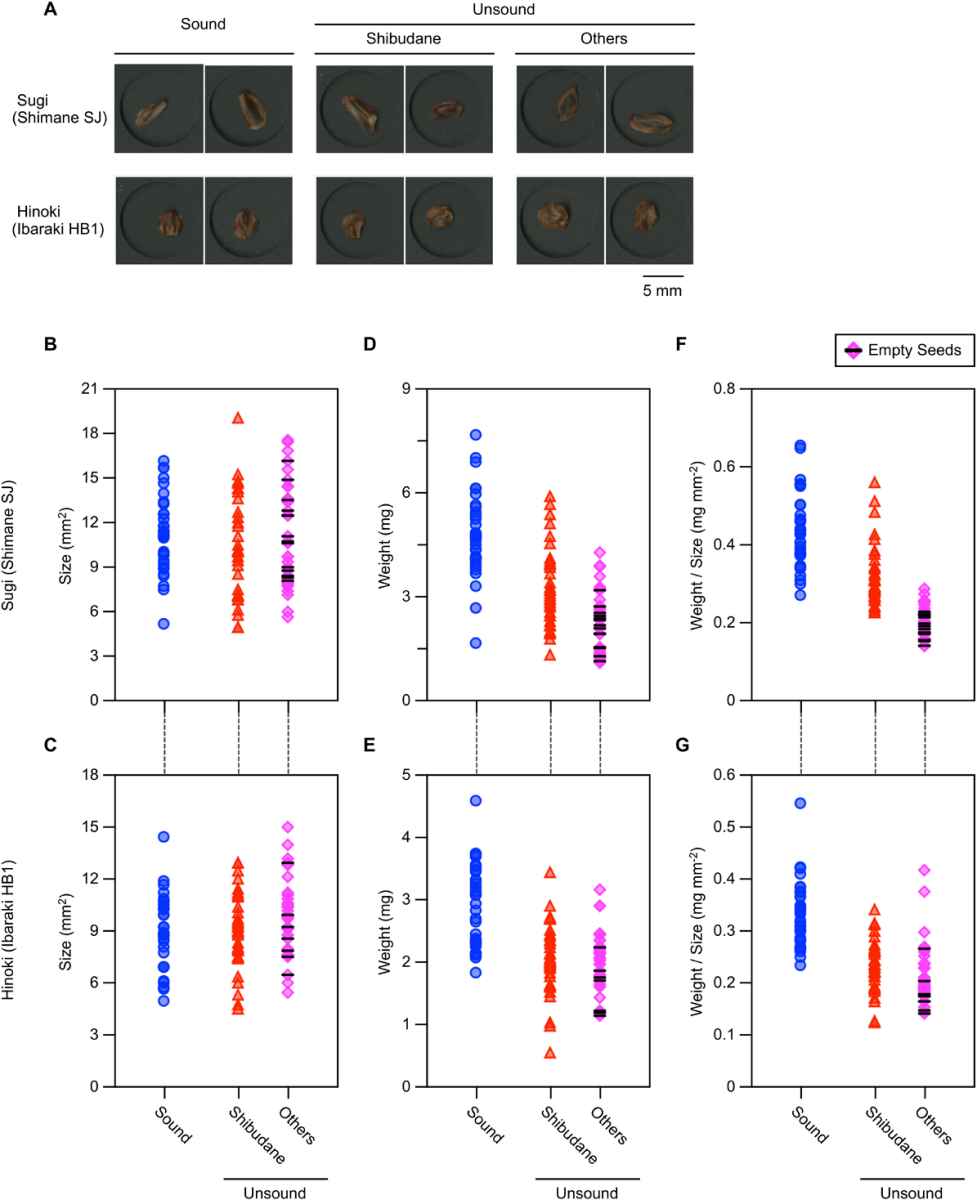
General features of sound and unsound seeds of *Cryptomeria japonica* (sugi) and *Chamaecyparis obtusa* (hinoki).

Among the 10 seed lots for each of sugi and hinoki, two for each were processed for image scanning in RGB format before subjected to a cutting test. Fig 3A compares the appearance of seeds that were later classified into three different groups. There appeared no visible features that helped us to estimate anatomical soundness of individual seeds. Size and weight of each seed group were also evaluated. As shown in Fig 3B to 3G, neither of these features served as effective measures to separate sound seeds out of unsorted seed lots. Although empty seeds, classified as others (highlighted by horizontal black bars in Fig 3B to 3G), were lighter in weight than sound seeds, especially when weight per unit size was compared (Fig 3F and 3G), they were quite rare in all seed lots examined in this study. Hence, exclusion of empty seeds by such means as sink/float and wind separations will not be an adequate solution to improve germination rate of sugi and hinoki seed lots.

### Spectroscopic features of sound and unsound sugi and hinoki seeds

**Fig 4:**
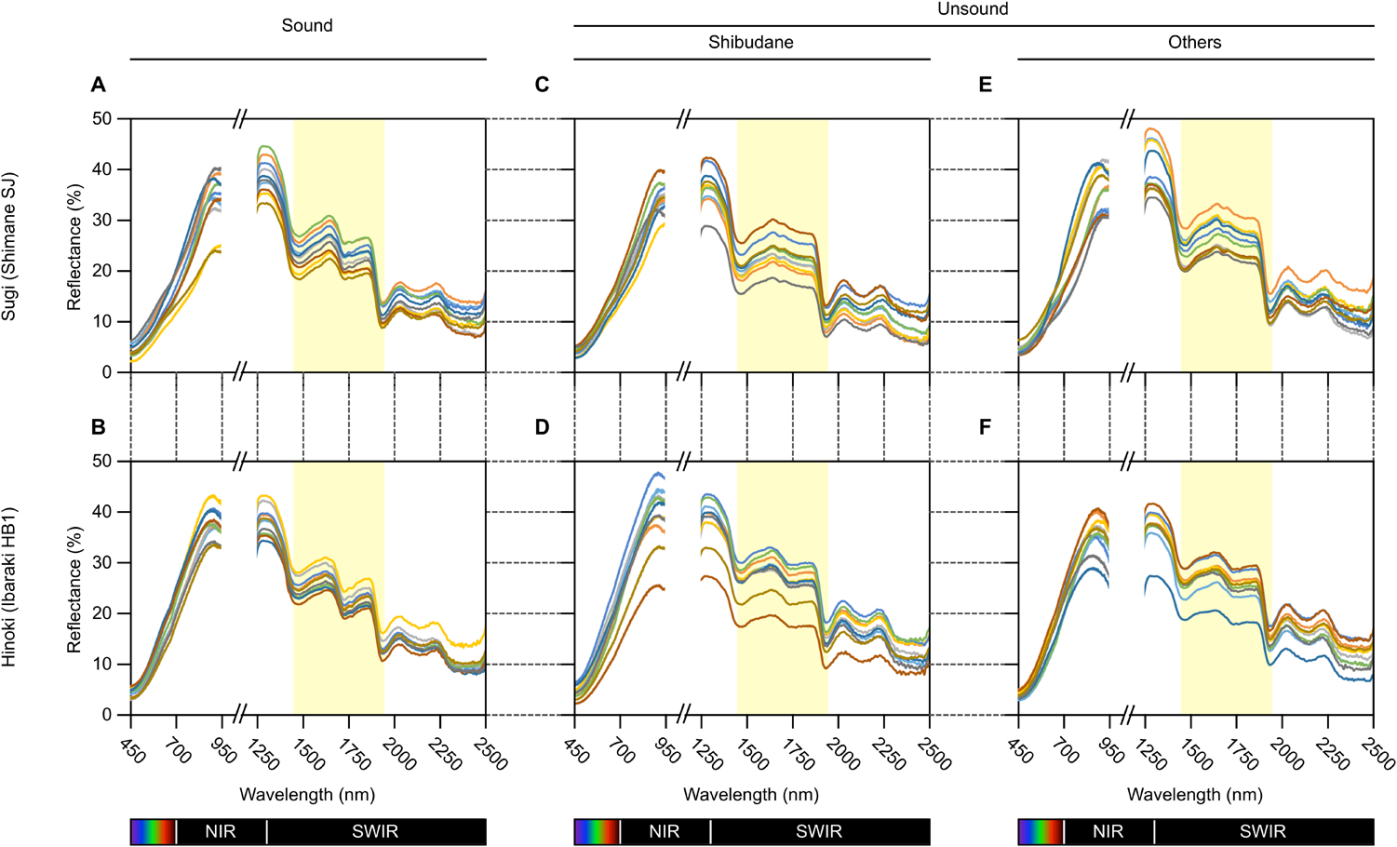
Spectroscopic features of sound and unsound seeds of *Cryptomeria japonica* (sugi) and *Chamaecyparis obtusa* (hinoki).

Although outer appearance of sound and unsound seeds in sugi and hinoki was indistinguishable by visible observation (Fig 3A), what is certain is that chemical composition inside should be more or less different. Given the infrared (IR) absorption is sensitive to chemical composition and structure, and the seed coat may have some permeability to IR light, it was expected whether anatomical soundness of individual seeds can be evaluated from their IR spectroscopic features. To test this hypothesis, the two seed lots for each tree species were, in fact, subjected to hyperspectral imaging in wavelength ranges from VIS to NIR and from NIR to SWIR prior to RGB imaging followed by a cutting test. Average reflectance spectra of the area corresponding to each individual seed were extracted from hyperspectral images using the software, SeedSpecAnalyzer (Fig 1A), which is provided as S1 File (see S1 Text for methods of operation). This software allows to highlight all pixels with reflectance spectra similar to those from a pre-assigned reference area (e.g., seed area) based on either method of cross correlation matching or spectral angle mapper, and then to auto-detect all closed areas of interest to calculate their corresponding reflectance spectra. Fig 4 shows representative spectra from 10 individual seeds each for the three different groups and for tree species. In the VIS/NIR range, no characteristic difference was observed in reflectance spectra among the three seed groups. In contrast, a conspicuous feature specific to sound seeds of both sugi and hinoki was identified in the SWIR spectral range; the reflectance in a narrow waveband centered at 1,730 nm was greatly depressed relative to that in adjacent wavebands on either side, representing “m-shaped” spectra (Fig 4A and 4B), compared to “n-shaped” (Fig 4C to 4F) that was common to both unsound seed groups.

Because the spectral depression in this SWIR waveband can be used as a measure to distinguish sound from unsound seeds, a reflectance index named SQI, which semi-quantifies the degree of the depression, was formulated as follows:

$$\alpha =\frac{\left(R_{1637}-R_{1734}\right)\times \left(R_{1854}-R_{1734}\right)-1}{\sqrt{{\left(R_{1637}-R_{1734}\right)}^{2}+1}\times \sqrt{{\left(R_{1854}-R_{1734}\right)}^{2}+1}}$$ (1)

$$\text{SQI}=\{\begin{array}{cc} \frac{180}{\pi }\times {\text{cos}}^{-1}\alpha , & \text{if}\;R_{1734}\leq \frac{R_{1637}+R_{1854}}{2}\\ 360-\frac{180}{\pi }\times {\text{cos}}^{-1}\alpha , & \text{otherwise} \end{array}$$ (2)

In these equations, *R*_*λ*_ represents reflectance in percent at wavelength λ. As illustrated in S1 Fig, SQI is equivalent to the clockwise angle in degree unit between two line segments (BA and BC in S1 Fig), and hence takes a positive value less than 360. It is expected that the lower the SQI of a seed (or its portion), the more it is likely to be sound.

### Single parameter method for sound sugi and hinoki seed selection

**Fig 5:**
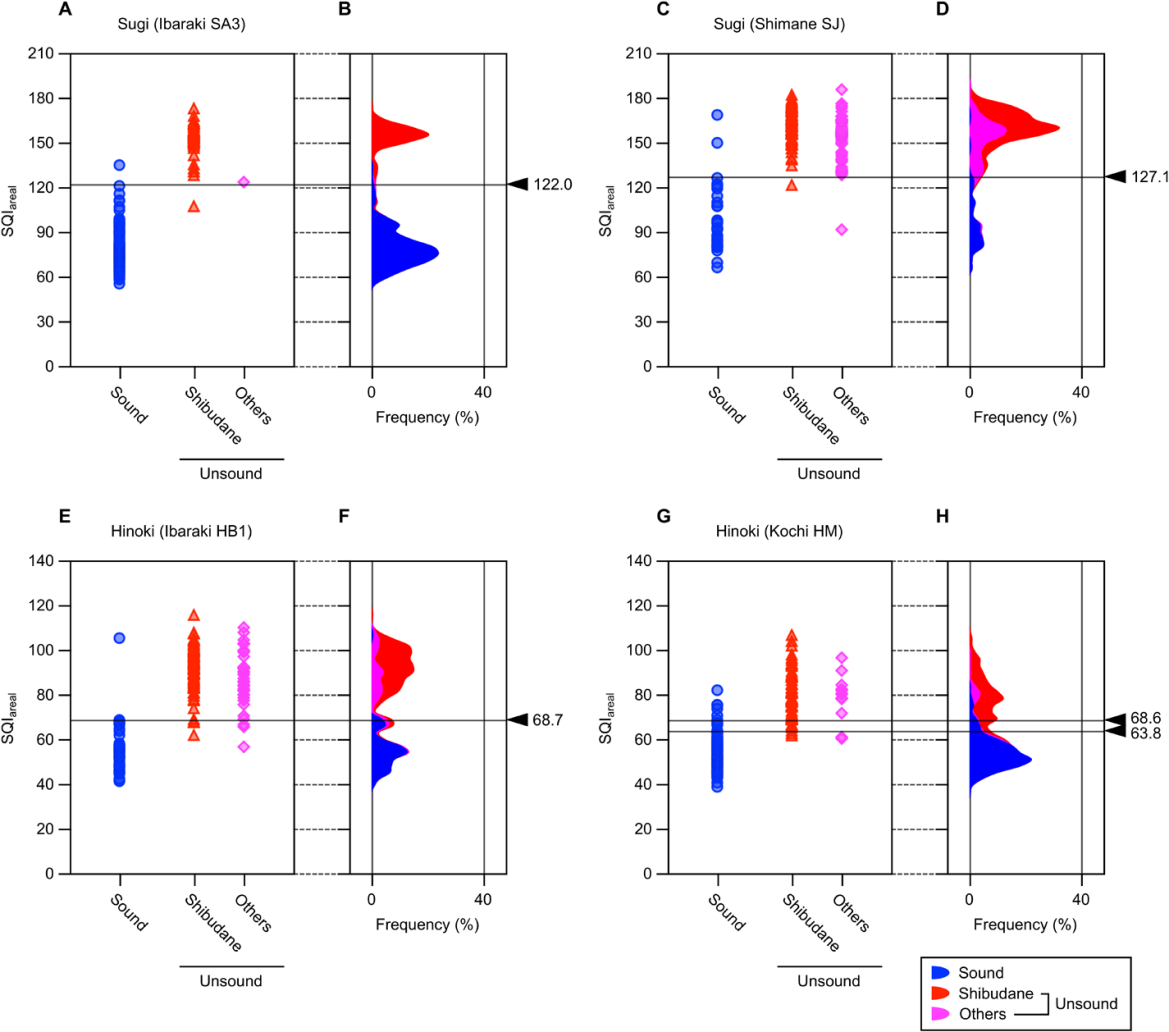
Correlation between anatomical soundness and seed quality index calculated from area-averaged reflectance spectra (SQI_areal_).

To ensure the reliability of SQI as a measure to estimate anatomical soundness of sugi and hinoki seeds, SQI was calculated for all individual seeds that have passed through hyperspectral imaging followed by a cutting test. Fig 5 shows distribution as well as frequency distribution of SQI for each seed group in the two seed lots for each tree species. Here, SQI was calculated from average reflectance spectra of the area corresponding to each individual seed. For clarity’s sake, SQI derived from area-averaged reflectance spectra will be hereafter expressed as SQI_areal,_ apart from SQI_pixel_ described later. Although distribution of SQI_areal_, especially the value range, was largely different between species, there appeared a tendency that sound seeds exhibited lower SQI_areal_ than unsound ones in every seed lot. If this was common to all sugi and hinoki seeds, it would be possible to schematize a procedure of sound seed selection by setting an upper threshold of SQI (*max*SQI) for each seed lot and then picking up all seeds representing SQI_areal_ lower than the *max*SQI. Because whether to select each individual seed is governed by a single parameter, *max*SQI, this seed selection procedure will be designated as “single parameter method”, apart from “dual parameter method” described later.

To evaluate the performance of sound seed selection, the following variables and statistics were defined. Consider a case where *N*_get_ out of total *N* seeds will be selected. The total seeds are composed of *N*_OK_ and *N*_NG_ (= *N* − *N*_OK_) sound and unsound seeds, respectively. At this stage, two statistics, initial sound seed rate (*SR*_init_) and recovery rate (*RR*), can be formulated as:

$$SR_{\text{init}}=\frac{N_{\text{OK}}}{N}$$ (3)

$$RR=\frac{N_{\text{get}}}{N}$$ (4)

The recovered *N*_get_ seeds can also be divided into *N*_get|OK_ and *N*_get|NG_ (= *N*_get_ − *N*_get|OK_) sound and unsound seeds, respectively. Then, three additional statistics, sound seed rate (*SR*), yield rate (of sound seeds) (*YR*), and total error rate (*TER*), which provide diagnostic measures of the seed selection performance, can be formulated as:

$$SR=\frac{N_{\text{get}|\text{OK}}}{N_{\text{get}}}\;\left(N_{\text{get}}>0\right)$$ (5)

$$YR=\frac{N_{\text{get}|\text{OK}}}{N_{\text{OK}}}\;\left(N_{\text{OK}}>0\right)$$ (6)

$$\begin{array}{r} TER=\frac{N_{\text{get}|\text{NG}}+N_{\text{OK}}-N_{\text{get}|\text{OK}}}{N}\\ =\frac{N_{\text{get}}+N_{\text{OK}}-N_{\text{get}|\text{OK}}\times 2}{N} \end{array}$$ (7)

**Fig 6:**
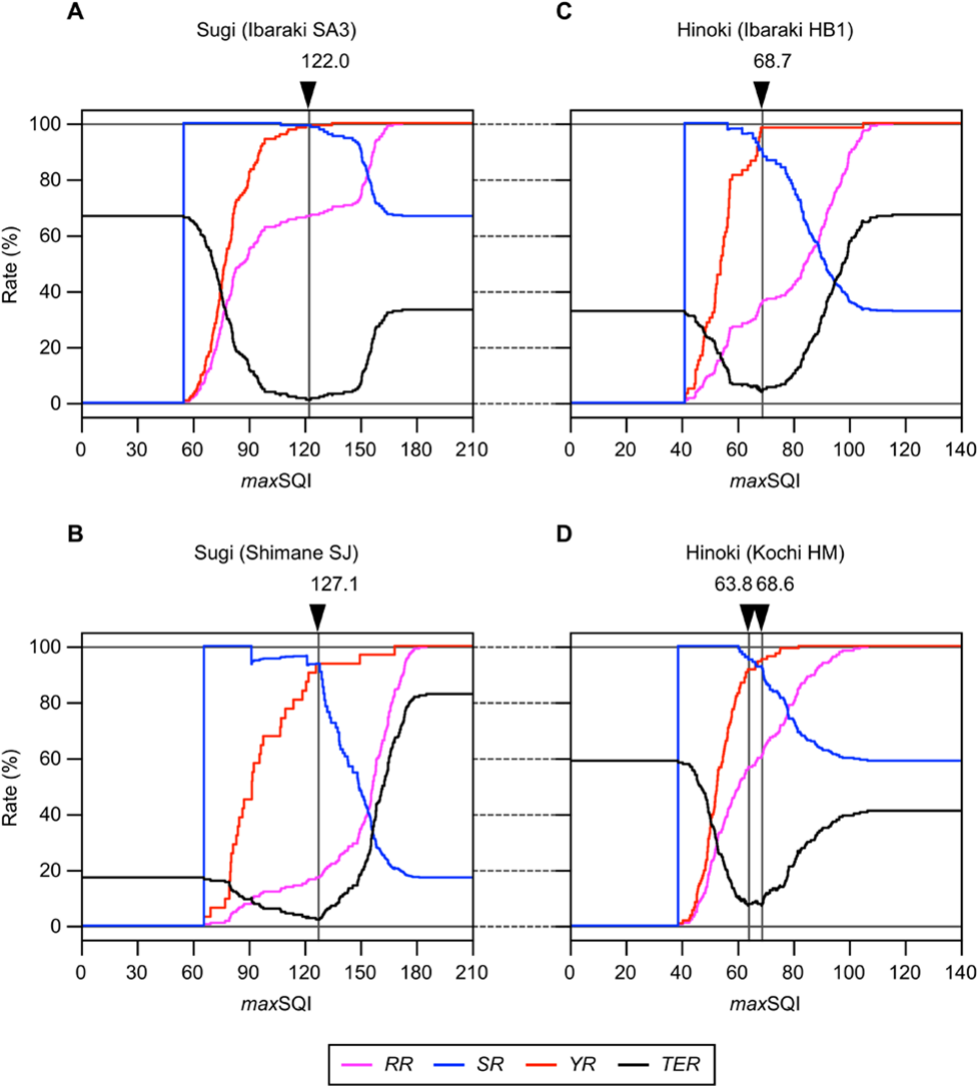
Statistics of sound seed selection by single parameter method.

The numerator of *TER* signifies the sum of the numbers of selected unsound and excluded sound seeds. In the single parameter method, *N*_get_ and *N*_get|OK_, and thereby *RR*, *SR*, *YR*, and *TER* as well, behave as functions of *max*SQI. Thus, the optimal value for this parameter can be searched for each seed lot from the correlation plots shown in Fig 6. For ideal selection, *SR* and *YR* should be maximized, or otherwise *TER* should be minimized. There is a constraint, however, that *SR* and *YR* cannot be maximized at the same time, because they have negative and positive correlations with *max*SQI, respectively. In contrast, *TER* shows a downward convex curve with its minimum value being found within a definite value range of *max*SQI. Thus, it is easier with *TER* to optimize *max*SQI, unless there is a specific requirement for sound seed selection, such as maximization of either *SR* or *YR* is preferred.

### Validation of single parameter method

As is evident from Fig 6, it is feasible to establish a seed lot of both sugi and hinoki that is composed of nearly 100% sound seeds (*SR* ≈ 1), if stringent selection was conducted with a low enough *max*SQI. In conditions where *TER* is minimized, both *SR* and *YR* still exceed 90% in every seed lot. However, what is essentially important is whether *max*SQI that was calibrated in a limited seed lot works similarly well in independent seed lots from either identical or different mother trees. To examine what we can call generalizing capability of the single parameter method, *max*SQI calibrated in one of the two seed lots of the same species by minimizing *TER* was validated in another seed lot in a pairwise fashion. Because the value range of SQI_areal_ was largely different between species (Fig 5), cross validity of *max*SQI across species was not considered here. As summarized in Table 2, minimum values of *TER* ranged from 1.1% to 7.2% for different seed lots, which were achieved when *max*SQI was set to 122.0 (seed lot Ibaraki SA3) and 127.1 (Shimane SJ) for sugi, and 68.7 (Ibaraki HB1) and either 63.8 or 68.6 (Kochi HM) for hinoki.

**Table 2:** Calibration and validation of *max*SQI in single parameter method for sound seed selection.

Species	Validation	Calibration			
***Cryptomeria japonica* (Sugi)**		**Seed Lot**	**Ibaraki SA3**	**Shimane SJ**	
		***SR***_**init**_ ^*a*^	66.7% (120/180)	17.2% (31/180)	
	**Seed Lot**	***max*SQI**	122.0	127.1	
	**Ibaraki SA3**	***RR*** ^*a*^	66.7% (120/180)	67.2% (121/180)	
		***SR*** ^*a*^	99.2% (119/120)	98.3% (119/121)	
		***YR*** ^*a*^	99.2% (119/120)	99.2% (119/120)	
		***TER*** ^*a*^	1.1% (2/180)^*b*^	1.7% (3/180)^*c*^	
	**Shimane SJ**	***RR*** ^*a*^	16.1% (29/180)	17.2% (31/180)	
		***SR*** ^*a*^	93.1% (27/29)	93.5% (29/31)	
		***YR*** ^*a*^	87.1% (27/31)	93.5% (29/31)	
		***TER*** ^*a*^	3.3% (6/180)^*c*^	2.2% (4/180)^*b*^	
***Chamaecyparis obtusa* (Hinoki)**		**Seed Lot**	**Ibaraki HB1**	**Kochi HM**	
		***SR***_**init**_ ^*a*^	32.8% (59/180)	58.9% (106/180)	
	**Seed Lot**	***max*SQI**	68.7	63.8	68.6
	**Ibaraki HB1**	***RR*** ^*a*^	35.6% (64/180)	28.9% (52/180)	35.6% (64/180)
		***SR*** ^*a*^	90.6% (58/64)	96.2% (50/52)	90.6% (58/64)
		***YR*** ^*a*^	98.3% (58/59)	84.7% (50/59)	98.3% (58/59)
		***TER*** ^*a*^	3.9% (7/180) ^*b*^	6.1% (11/180)^*c*^	3.9% (7/180)^*c*^
	**Kochi HM**	***RR*** ^*a*^	60.6% (109/180)	56.1% (101/180)	60.6% (109/180)
		***SR*** ^*a*^	92.7% (101/109)	96.0% (97/101)	92.7% (101/109)
		***YR*** ^*a*^	95.3% (101/106)	91.5% (97/106)	95.3% (101/106)
		***TER*** ^*a*^	7.2% (13/180)^*c*^	7.2% (13/180)^*b*^	

^*a*^ For definition of *SR*_init_, *RR*, *SR*, *YR* and *TER*, see Eqs 1–7.

^*b*^ Calibration errors.

^*c*^ Validation errors.

Here a case of calibration and validation of *max*SQI performed with a sugi seed lot, Ibaraki SA3, is demonstrated. This lot consisted of 180 seeds (= *N*), of which 120 (= *N*_OK_) turned out to be sound by a cutting test (*SR*_init_ = 120/180). If *max*SQI of 122.0 was applied to the “self” seed lot, 120 seeds (= *N*_get_) are recovered after selection (*RR* = 120/180), of which 119 (= *N*_get|OK_) are sound seeds (hence both *SR* and *YR* equal 119/120). Because the remaining one recovered seed is unsound shibudane, and one sound seed is excluded from the selection, the total number of misclassified seeds is two. This is the condition where *TER* in this seed lot takes its minimum value of 1.1% (2/180), which can be interpreted as the calibration error. Then the same *max*SQI will be applied for validation to another seed lot of sugi, Shimane SJ. The value 122.0 appeared more stringent than *max*SQI calibrated in this seed lot (127.1). Actually, compared to the case where *max*SQI of 127.1 was applied, the number of recovered seeds becomes lower by two (*N*_get_ = 29), both of which are sound seeds (*N*_get|OK_ = 27). Even though, the resulting *TER* is still as low as 3.3% (6/180), which can be interpreted as the validation error. For all four cases of validation, *TER* did not exceed 7.2%, the highest (worst) value of the calibration errors. Thus, once *max*SQI was calibrated in a single seed lot, the same value will work quite well in independent seed lot of the same species.

### Spatial distribution of SQI in sound and unsound sugi and hinoki seeds

As described above, SQI_areal_ for each individual seed was calculated from average reflectance spectra of its corresponding area in the SWIR hyperspectral image. In averaging reflectance spectra, the software SeedSpecAnalyzer allows to exclude pixels that are located within a fixed margin from the contour of the seed area (S2A and S2B Fig). This primarily aimed at concentrating on pixels that purely reflect spectroscopic features of seeds, not being disturbed by the background spectra. Serendipitously, this led to identification of spatial nonuniformity of SQI within seeds. S2C to S2F Fig shows correlations between the margin setting and either distribution or frequency distribution of SQI_areal_ for each seed group in seed lots, Shimane SJ for sugi and Ibaraki HB1 for hinoki. In sound seeds of both species, SQI_areal_ decreased as the averaging area was narrowed toward the center by increasing the margin setting. In contrast, the response of SQI_areal_ in unsound seeds was different between species; while SQI_areal_ in unsound hinoki seeds was negative proportional to the margin setting as in sound seeds, there appeared no such correlation in unsound sugi seeds.

**Fig 7:**
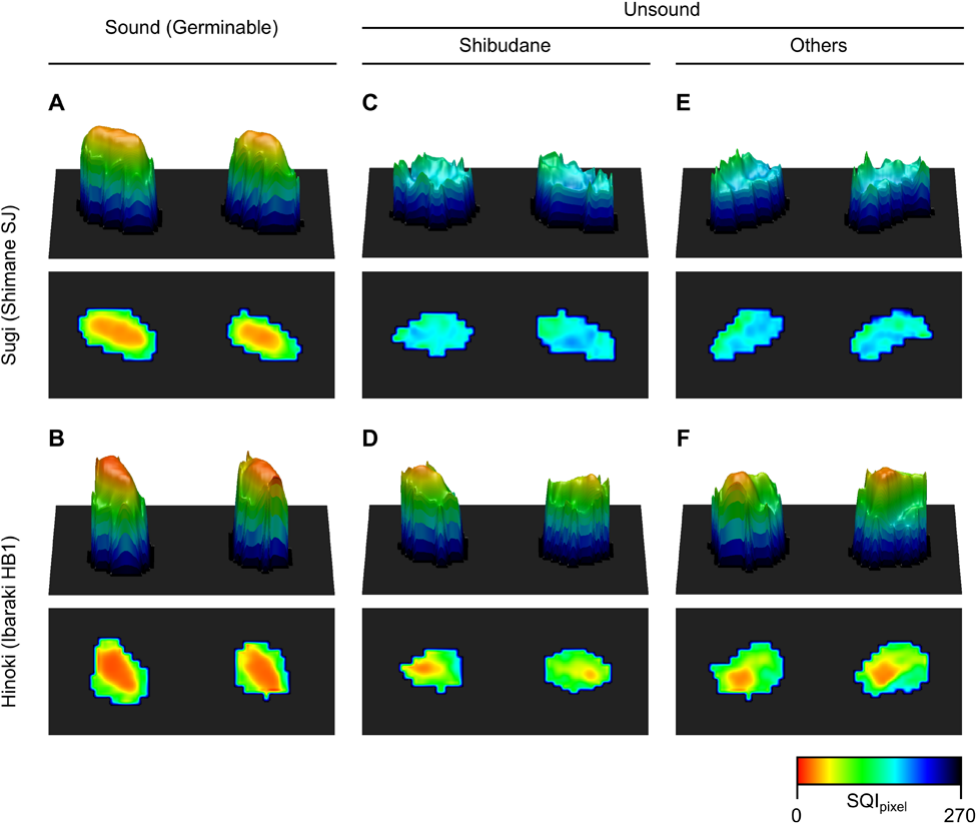
Spatial distribution of pixelwise seed quality index (SQI_pixel_) in sound and unsound seeds of *Cryptomeria japonica* (sugi) and *Chamaecyparis obtusa* (hinoki).

To investigate the basis of these observations, another software, SQIViewer, allowing to visualize and export spatial distribution data of SQI was constructed (Fig 1B and 1C), which is also packaged in S1 File (see S1 Text for methods of operation). Because each pixel in hyperspectral images contains spectral reflectance information, it is possible to calculate SQI in a pixelwise fashion. To distinguish it from SQI_areal_, the pixelwise SQI will be designated as SQI_pixel_. The software is also equipped with an option of distance-weighted smoothing filter (3 × 3 pixel size, the weight is set to two to the negative power of the distance), so that reflectance spectra from a single pixel can be denoised before calculating SQI_pixel_. In the following results, however, this option was used only for the purpose of drawing Fig 7. This figure illustrates spatial distribution patterns of SQI_pixel_ in each group of sugi and hinoki seeds in the forms of heat maps and three-dimensional plots. The data are consistent with the correlation between SQI_areal_ and the margin setting (S2C to S2F Fig); lower SQI_pixel_ values are localized around the center of sound seeds and unsound hinoki seeds (Fig 7A, 7B, 7D and 7F), whereas there is no such spatial gradient of SQI_pixel_ in unsound sugi seeds (Fig 7C and 7E). The figure also corroborates the lower value ranges of SQI_areal_ in all groups of seeds in hinoki than in sugi (see Fig 5 and S2C to S2F Fig).

### Dual parameter method for sound sugi and hinoki seed selection

Because value ranges of SQI_areal_ in sound and unsound seeds were not separated as clearly in hinoki as in sugi (Fig 5 and S2C to S2F Fig), sound seed selection by the single parameter method worked less accurately in hinoki (see Table 2). Hence, it was questioned whether spatial distribution patterns of SQI_pixel_ can help to improve the accuracy of sound hinoki seed selection. Based on the comparison between distribution patterns of SQI_pixel_ in sound and unsound hinoki seeds (Fig 7B, 7D and 7F), the ratio of low SQI_pixel_ area (drawn in warm colors) to the total seed area appeared larger in sound seeds. This area ratio is defined as PBR (for peak-to-base area ratio), where “peak area” refers to the area occupied by pixels representing SQI_pixel_ lower than *max*SQI. Like SQI_areal_, PBR can be calculated for every individual seed.

By setting a lower threshold of PBR (*min*PBR) in addition to *max*SQI, another procedure for sound seed selection, designated as “dual parameter method”, can be schematized. In this method, PBR behaves as a dependent variate of *max*SQI, while whether to select each individual seed depends on the magnitude relation between PBR and *min*PBR; seeds representing PBR greater than *min*PBR are considered as sound. The performance of sound seed selection by this method can be again evaluated using the statistics, *RR*, *SR*, *YR*, and *TER*, which are analogous to those introduced for the single parameter method (Eqs 4–7). In the current case, however, *N*_get_ and *N*_get|OK_, and thereby the above statistics behave as functions of dual parameters, *max*SQI and *min*PBR.

**Fig 8:**
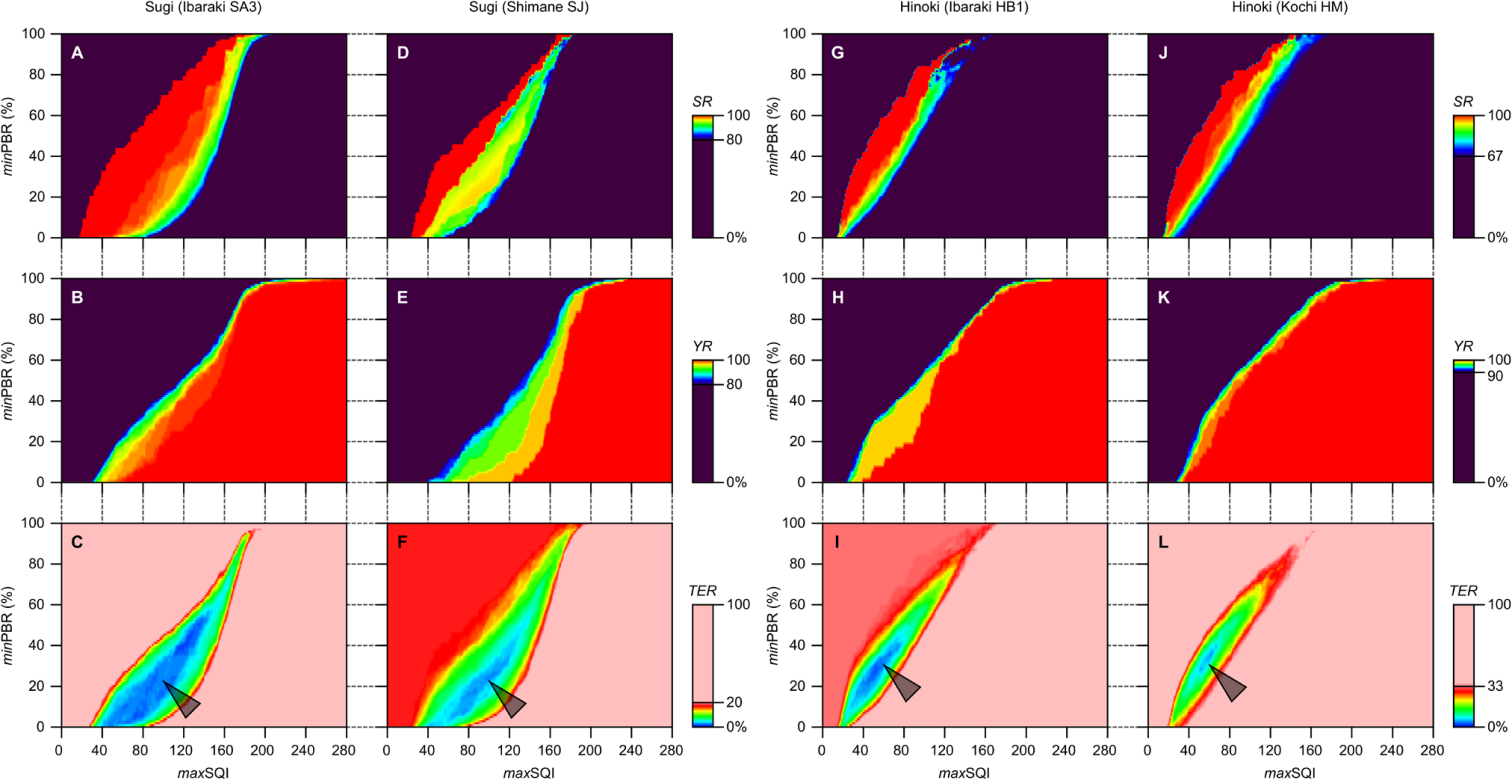
Statistics of sound seed selection by dual parameter method.

The correlations between these parameters and either of *SR*, *YR*, or *TER* in each seed lot are shown as heat maps in Fig 8. The heat maps for each statistics look very similar, especially when those for independent seed lots of the same species are compared. The optimal dual parameters can be searched for each seed lot from any of the three heat maps. Yet, it is easiest with *TER* maps (Fig 8C, 8F, 8I and 8L) to single out an optimal parameter pair, because candidates of such dual parameters are enclosed in a small heat map area shaped like “inner flame of a burning candle” (indicated by arrowheads in Fig 8). One or two of optimal parameter pairs for each seed lot is shown in Table 3.

**Table 3:** Calibration and validation of *max*SQI and *min*PBR in dual parameter method for sound seed selection.

Species	Validation	Calibration			
***Cryptomeria japonica* (Sugi)**		**Seed Lot**	**Ibaraki SA3**	**Shimane SJ**	
		***SR***_**init**_ ^*a*^	66.7% (120/180)	17.2% (31/180)	
		***max*SQI**	125	88	
	**Seed Lot**	***min*PBR**	40%	17%	
	**Ibaraki SA3**	***RR*** ^*a*^	66.7% (120/180)	66.7% (120/180)	
		***SR*** ^*a*^	99.2% (119/120)	98.3% (118/120)	
		***YR*** ^*a*^	99.2% (119/120)	98.3% (118/120)	
		***TER*** ^*a*^	1.1% (2/180)^*b*^	2.2% (4/180) ^*c*^	
	**Shimane SJ**	***RR*** ^*a*^	16.1% (29/180)	16.1% (29/180)	
		***SR*** ^*a*^	93.1% (27/29)	96.6% (28/29)	
		***YR*** ^*a*^	87.1% (27/31)	90.3% (28/31)	
		***TER*** ^*a*^	3.3% (6/180)^*c*^	2.2% (4/180)^*b*^	
***Chamaecyparis obtusa* (Hinoki)**		**Seed Lot**	**Ibaraki HB1**	**Kochi HM**	
		***SR***_**init**_ ^*a*^	32.8% (59/180)	58.9% (106/180)	
		***max*SQI**	53	53	63
	**Seed Lot**	***min*PBR**	27%	29%	36%
	**Ibaraki HB1**	***RR*** ^*a*^	33.9% (61/180)	33.3% (60/180)	28.9% (52/180)
		***SR*** ^*a*^	95.1% (58/61)	95.0% (57/60)	98.1% (51/52)
		***YR*** ^*a*^	98.3% (58/59)	96.6% (57/59)	86.4% (51/59)
		***TER*** ^*a*^	2.2% (4/180)^*b*^	2.8% (5/180)^*c*^	5.0% (9/180)^*c*^
	**Kochi HM**	***RR*** ^*a*^	61.1% (110/180)	57.2% (103/180)	59.4% (107/180)
		***SR*** ^*a*^	92.7% (102/110)	98.1% (101/103)	96.3% (103/107)
		***YR*** ^*a*^	96.2% (102/106)	95.3% (101/106)	97.2% (103/106)
		***TER*** ^*a*^	6.7% (12/180)^*c*^	3.9% (7/180)^*b*^	

^*a*^ For definition of *SR*_init_, *RR*, *SR*, *YR* and *TER*, see Eqs 1–7.

^*b*^ Calibration errors.

^*c*^ Validation errors.

### Validation of dual parameter method

Here a case of calibration and validation of dual parameters performed with a hinoki seed lot, Ibaraki HB1, is demonstrated. When a grid search for minimum *TER* was conducted at intervals of one for *max*SQI and 1% for *min*PBR, 16 pairs were identified as optimal dual parameters. The values shown in Table 3, 53 and 27% for *max*SQI and *min*PBR, respectively, were chosen as they were modes of the 16 candidates for each parameter. In case this parameter pair was applied to the “self” seed lot, 61 (= *N*_get_) out of 180 seeds in total (= *N*) are recovered after selection (*RR* = 61/180), of which 58 (= *N*_get|OK_) are sound seeds (*SR* = 58/61). In more detail, one out of 59 sound seeds in this seed lot (= *N*_OK_) is excluded from the selection (*YR* = 58/59), while three unsound seeds, one classified as shibudane and two as others, escape from exclusion. Thus, the total number of misclassified seeds is four. This is one of the conditions where *TER* in this seed lot takes its minimum value of 2.2% (4/180), which can be interpreted as the calibration error. Then the same dual parameters will be applied for validation to another seed lot of hinoki, Kochi HM. Apparently, they are close to one of two calibrated parameter pairs in Kochi HM seed lot; 53 and 29% for *max*SQI and *min*PBR, respectively, with which *TER* is minimized to 3.9% (7/180). The suboptimal lower *min*PBR (27%), however, should reduce the stringency of sound seed selection. Actually, the number of recovered seeds becomes greater by seven (*N*_get_ = 110), due to the lower *min*PBR with an absolute difference of 2%. Up to six out of the seven seeds are unsound, which are further classified into five shibudane and one others. Together, the dual parameters calibrated in Ibaraki HB1 seed lot achieve *TER* of 6.7% (12/180) in Kochi HM seed lot. This *TER*, interpreted as the validation error, is not as low as the calibration error (2.2%). Yet, considering the lower overall values of *TER* in Table 3 compared to those in Table 2, dual parameter method should be better suited for sound hinoki seed selection than single parameter method. It is important, however, to notice that calibration of optimal dual parameters requires far more seeds than in single parameter method. With each seed lot examined being equally composed of 180 seeds, the parameters shown in Table 3 could be still suboptimal.

The software SQIViewer is compatible with sound seed selection based on the dual parameter method. By setting appropriate values for *max*SQI and *min*PBR, seeds that are presumed as sound are highlighted with a saturated color (Fig 1C).

### Sound seed selection improved germination rate of sugi and hinoki seeds

The further aim beyond developing the methods for sound seed selection is to produce sugi and hinoki seed lots with a high germination rate. To ensure the effectiveness of sound seed selection on improving germination rate, another 180 seeds were drawn from each of the two sugi and two hinoki seed lots, and subjected to a germination test after evaluating anatomical soundness of each individual seed via SWIR hyperspectral imaging. At the end of the test period or just after germination, seeds were further examined by a cutting test. Once seeds were imbibed, however, it became difficult to distinguish between sound and underdeveloped endosperm based on the cross sectional color and appearance. On the other hand, shibudane was easy to distinguish from the others. Because none of shibudane germinated at all, seeds were classified here into the following three groups; germinable, shibudane, and other ungerminated seeds. As already shown in Table 1, germination rate of each seed lot had declined to a considerable extent during long-term storage since it was initially determined after the seed harvesting (ranging from 0.5 to 5 years). Hence, seeds classified as “other ungerminated” should contain a certain proportion of sound seeds. It is also presumable that germinable seeds should be a subset of sound seeds.

**Fig 9:**
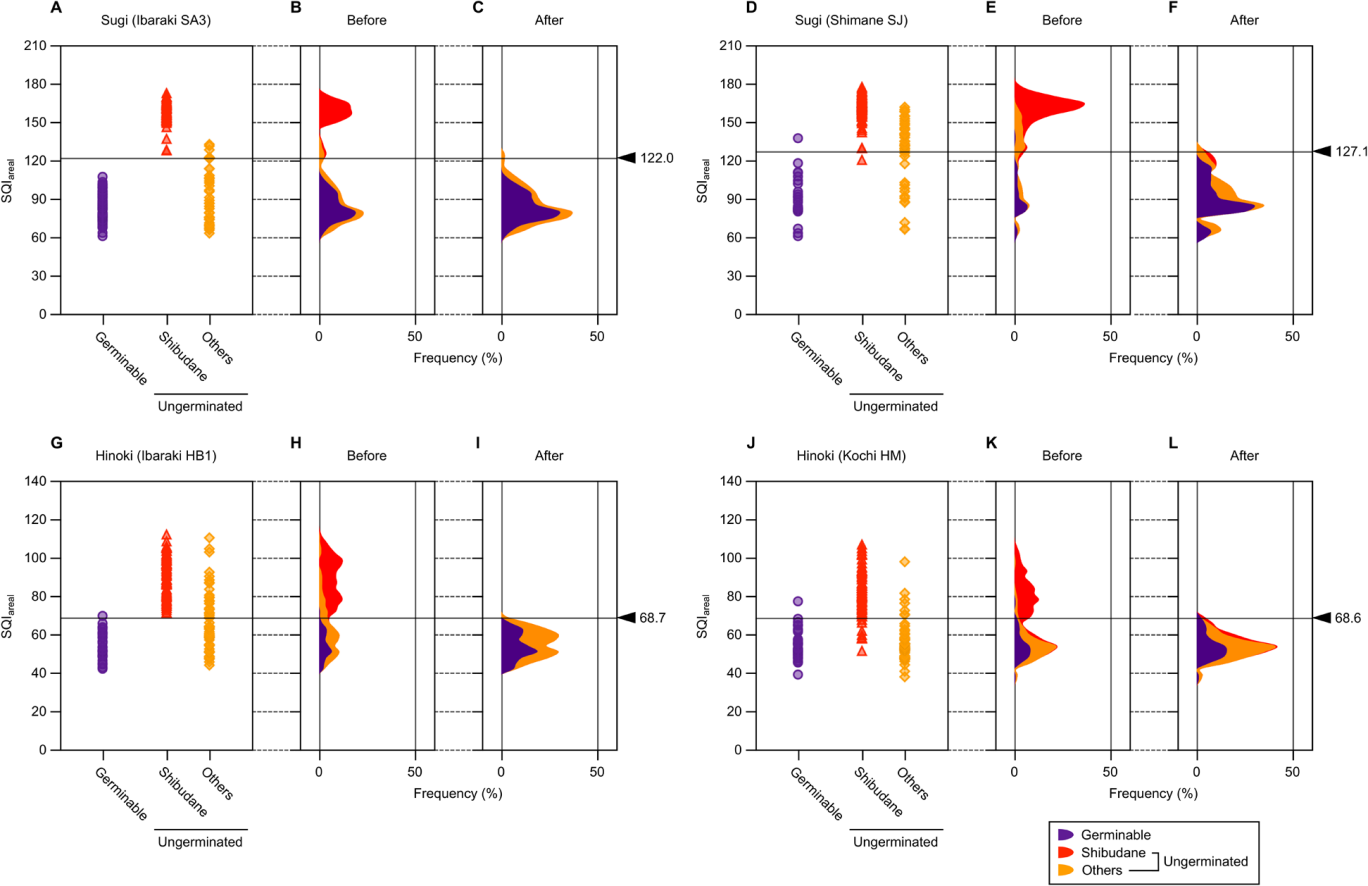
Effects of single parameter method for sound seed selection on improving germination rate of *Cryptomeria japonica* (sugi) and *Chamaecyparis obtusa* (hinoki) seed lots.

Fig 9 illustrates how single parameter method contributed to increase the proportion of germinable seeds. Further details regarding germination rate of each seed lot before and after sound seed selection by either single or dual parameter method are summarized in Table 4. Selection with parameters optimized for each seed lot did not result in severe loss of germinable seeds, while nearly all shibudane was excluded efficiently. In contrast, it is uncertain whether each individual seed classified as “other ungerminated” was viable, i.e., maintained a capacity to germinate upon exposure to favorable stimuli or environment. This is because in order for a seed to be tested for its germinability, the viability at the time of sowing need to be evaluated in a nondestructive fashion, which remains to be established for sugi and hinoki seeds.

**Table 4:** Germination rate of *Cryptomeria japonica* and *Chamaecyparis obtusa* seed lots before and after sound seed selection.

**Species**			***Cryptomeria japonica* (Sugi)**					
**Seed Lot**			**Ibaraki SA3**			**Shimane SJ**		
**Initial Germination Rate**			62% (May 2012)			18.3% (Feb. 2014)		
**Selection Method**			None	Single	Dual	None	Single	Dual
**Parameters**	***max*SQI**		-	122.0	125	-	127.1	88
	***min*PBR**		-	-	40%	-	-	17%
**Seed Group**	**Germinable**		43.9% (79/180)	70.5% (79/112)	69.9% (79/113)	14.4% (26/180)	62.5% (25/40)	67.6% (25/37)
	**Ungerminated**	**Shibudane**	35.0% (63/180)	0.0% (0/112)	0.9% (1/113)	62.8% (113/180)	2.5% (1/40)	0.0% (0/37)
		**Others**	21.1% (38/180)	29.5% (33/112)	29.2% (33/113)	22.8% (41/180)	35.0% (14/40)	32.4% (12/37)
	**Total**		100.0% (180/180)	100.0% (112/112)	100.0% (113/113)	100.0% (180/180)	100.0% (40/40)	100.0% (37/37)
**Species**			***Chamaecyparis obtusa* (Hinoki)**					
**Seed Lot**			**Ibaraki HB1**			**Kochi HM**		
**Initial Germination Rate**			41% (Jun. 2012)			53% (Jun. 2009)		
**Selection Method**			None	Single	Dual	None	Single	Dual
**Parameters**	***max*SQI**		-	68.7	53	-	68.6	53
	***min*PBR**		-	-	27%	-	-	29%
**Seed Group**	**Germinable**		17.2% (31/180)	46.9% (30/64)	46.9% (30/64)	21.1% (38/180)	38.6% (37/96)	39.8% (37/93)
	**Ungerminated**	**Shibudane**	51.1% (92/180)	0.0% (0/64)	0.0% (0/64)	46.7% (84/180)	8.3% (8/96)	5.4% (5/93)
		**Others**	31.7% (57/180)	53.1% (34/64)	53.1% (34/64)	32.2% (58/180)	53.1% (51/96)	54.8% (51/93)
	**Total**		100.0% (180/180)	100.0% (64/64)	100.0% (64/64)	100.0% (180/180)	100.0% (96/96)	100.0% (93/93)

To estimate the viability of sound seeds in each seed lot, 24 sound seeds selected from each lot were evaluated by tetrazolium staining for cellular respiration activity. For sugi, all 24 seeds each from the two independent seed lots exhibited strong red color in the embryo, suggesting that all of them were viable. In contrast, there were unstained sound seeds in hinoki seed lots; visible red color in the embryo was observed only in 20 (83.3%) and 10 (41.7%) seeds from Ibaraki HB1 and Kochi HM seed lots, respectively. This suggests that the seed storage condition applied in this study was not necessarily optimal for hinoki seeds. Nevertheless, unless all seeds had become inviable, sound seed selection can improve germination rate of both sugi and hinoki seed lots.

### Soundness of sugi seeds is associated with the abundance of fatty acids

Given SQI acts as an indicator of anatomical soundness of sugi and hinoki seeds, it is questioned which biochemical features are sensed by this reflectance index. As described above, SQI was formulated as the measure of spectral depression in the reflectance around 1,730 nm (S1 Fig). Lipids are one of the major constituents in seeds that can account for both germinability and spectral depression in the SWIR waveband. To confirm whether the abundance of lipids is associated with seed groups, lipophilic extracts from each group of sugi and hinoki seeds were subjected to GC-MS analysis. Table 5 shows the major lipophilic compounds and their relative composition in sound seeds, shibudane, and other unsound seeds of each tree species. The data obtained from sugi seeds were quite simple to interpret; in sound seeds, fatty acids, possibly released form glycerides, accounted for as much as three quarters of the total lipophilic compounds. In contrast, their composition was less than one quarter in both groups of unsound seeds; instead, the decrease in fatty acids was largely compensated by the accumulation of substances such as 4-(thian-4-yl)thiane and diterpenes, isopimaric acid and ferruginol, which were almost absent in sound seeds. On the other hand, composition of lipophilic compounds in hinoki seeds was far more complex than in sugi seeds. The most abundant molecular species detected in all hinoki seed groups were diterpene ketophenol, totarolone and its allies, which accounted for nearly one quarter of the total lipophilic compounds. Another major compounds were 4-(thian-4-yl)thiane and ferruginol, which, in hinoki, were detected also in sound seeds, while isopimaric acid was absent in all seed groups. Surprisingly, fatty acids accounted for only 1.5% of the total lipophilic compounds in sound hinoki seeds, and there was no apparent difference in their composition between sound and unsound “non-shibudane” seeds.

**Table 5:** Composition of the major lipophilic compounds in different seed groups of *Cryptomeria japonica* and *Chamaecyparis obtusa*.

Species (Seed Lot)		Relative Composition (%)			
		Seed Group			
			Unsound		
	Compound	Sound	Shibudane	Others	Hit Rate ^*a*^ (%)
***Cryptomeria japonica* (Sugi, Shimane SJ)**	Fatty acids (C16, C18)	72.5	23.9	17.7	≥ 98
	Long-chain hydrocarbons	7.3	8.5	2.7	≥ 91
	Isopimaric acid	0.9	12.5	12.2	≥ 96
	4-(Thian-4-yl)thiane	0.0	14.0	14.8	≥ 91
	Ferruginol	0.0	2.0	1.9	≥ 99
***Chamaecyparis obtusa* (Hinoki, Ibaraki HB1)**	Totarolone (13-Hydroxy-14-isopropylpodocarpa-8,11,13-trien-3-one)	16.5	18.4	20.5	≥ 93
	Ferruginol	9.1	11.4	12.5	≥ 83
	6-Hydroxy-1,1,4a-trimethyl-7-propan-2-yl-10,10a-dihydro-9H-phenanthren-2-one	6.4	6.4	5.6	≥ 83
	4-(Thian-4-yl)thiane	5.7	6.1	5.9	≥ 90
	Monoterpenes	5.2	2.2	2.7	≥ 91
	Fatty acids (C16, C18)	1.5	0.4	1.5	≥ 99

^*a*^ Minimal hit rate obtained from database searches using GC-MS spectrogram from either of the three seed groups.

The intense light absorption by biologic samples at 1,730 nm can be ascribed to the first overtone of stretching vibration of carbon-hydrogen bonds, which are abundant in fatty acid moieties of lipids. However, it would be improvident to conclude that SQI acts as the lipid sensor, because absorption spectral data for each of the major constituents in sugi and hinoki seeds are not fully available. At least in sugi, it appears that high accumulation of fatty acids should be essential for seeds to germinate, and that this contributed to some extent to depress the reflectance around 1,730 nm in sound sugi seeds (Fig 4A).

## Discussion

In this study, we developed methods for selecting sound seeds of coniferous tree species, sugi and hinoki. Although the methods are based on hyperspectral imaging in the SWIR range, measuring reflectance in a total of 163 wavebands, only images in three narrow wavebands close with each other and containing 1,730 nm at the center were sufficient to evaluate anatomical soundness of seeds in these two species (Fig 4 and S1 Fig). After selection of sound seeds, germination rate of each seed lot did increase to a considerable extent, but did not reach 100% (Fig 9 and Table 4). This might be due in part to misclassification of unsound seeds as being sound, but largely to inclusion of ungerminated sound seeds. Failure of these sound seeds to germinate within the test period could be attributed to dormancy, suboptimal culture conditions, and/or, especially for hinoki seeds, loss of viability. We have yet to succeed in discriminating germinable and ungerminated sound seeds using reflectance in the three wavebands and also in all of 163 wavebands combined with multivariate machine learning techniques. This is possibly because only small number of reflectance spectra for these two seed groups could have been analyzed (e.g., at most 37 germinable and 51 ungerminated sound seeds in Kochi HM seed lot; see Table 4) or otherwise reflectance spectra of these two seed groups were too close to be distinguished from one another. In any case, it will be of critical importance toward achieving high germination rate with sugi and hinoki seed lots to further optimize the culture conditions and to elaborate a method of presowing treatment for breaking dormancy. For hinoki seeds, it is also essential to identify optimal conditions for long-term storage.

In classifying sound seeds of sugi and hinoki, we did not depend on multivariate chemometric approaches such as principal component analysis (PCA) and partial least squares-discriminant analysis (PLS-DA), as have been used to discriminate viable seeds in wheat, barley and sorghum [13]. This is primarily because, despite similarity in outer appearance (Fig 3A), inner structure and possibly chemical composition of sound and unsound seeds were largely different from each other (Fig 2A to 2F), and the difference could be readily appreciable through their spectroscopic features in the SWIR range (Fig 4). More importantly, however, the small number of wavebands required for sound seed selection allow an opportunity to develop low-cost and easy-to-use imaging instruments optimized for this specialized purpose; a general SWIR camera attached to a filter wheel with specific narrow bandpass filters can substitute for expensive and complex hyperspectral systems.

Although still preliminary, quantification of lipophilic compounds in sound and unsound sugi and hinoki seeds by GC-MS brought intriguing results in understanding why reflectance spectra in the SWIR range were different between these seed groups and in speculating biological relevance of the formation of shibudane and the wide difference in chemical composition between sugi and hinoki seeds (Table 5). In sugi, higher abundance of fatty acids in sound seeds than in unsound seeds may be the cause of lower reflectance (higher light absorption) around 1,730 nm. In contrast, isopimaric acid and ferruginol, which have been reported to possess antimicrobial activities [14], were accumulated exclusively in unsound seeds. Because shibudane is specific to Cupressaceae family trees and its inner morphology is fairly uniform among different particles, generally high but differing rates of shibudane formation (Table 1) could have some bearing on its potential but as yet unexplored role in defense against bacterial attack. In hinoki seeds, by contrast, the most abundant lipophilic compounds were totarolone and its allies, which are related to totarol known to inhibit bacterial proliferation by targeting FtsZ [15]. It was noticed during a cutting test that hinoki seeds, including shibudane, emit strong aromatic odor when their basal portion was bisected. Actually, they possessed organs like honey stomach outside the endosperm, which were filled with aromatic liquid. It could be that most of the major lipophilic compounds in hinoki seeds that are absent in sugi seeds are present as such extra-endospermic components and do not directly involved in germination. Such differences in structure and chemical composition could have brought about faster viability loss in hinoki than in sugi seeds, and thus will provide a clue in identifying optimal storage conditions for hinoki seeds. Interestingly, ferruginol that was undetectable in sound sugi seeds was the second most abundant lipophilic compound in sound hinoki seeds (Table 5). This implies contrasting antimicrobial strategies of seeds between these two species; while hinoki pours in massive resources to produce and protect a single seed, sugi does not but instead differentiates the role of sound seeds and unsound shibudane for reproduction and defense, respectively.

Although chemical compounds that act as immediate markers of sound hinoki seeds could not be determined, reflectance spectra in the SWIR range were no less useful for sound seed selection. Because this is likely attributable to indirect detection of endospermic compounds, in-depth analysis of reflectance spectra in comparison with chemical composition of different seed groups will contribute to expand the versatility and to improve the precision of sound seed selection based on narrow-multiband spectral imaging. The methods presented here will be soon put into practical use in producing container seedlings for reforestation in Japan.

## Supporting Information

S1 Fig Geometric interpretation of seed quality index (SQI). (TIF)

S2 Fig Correlation between seed quality index (SQI_areal_) value and exclusion border width in individual seeds of *Cryptomeria japonica* (sugi) and *Chamaecyparis obtusa* (hinoki). (TIF)

S1 File Installer of hyperspectral image analysis software and sample image files of *Cryptomeria japonica* (sugi) and *Chamaecyparis obtusa* (hinoki) seeds. (ZIP)

S1 Text Legends to S1 File. (DOCX)

## References

[1] Fujitake I (2007) Selection of tree species for plantations in Japan. Forest Policy and Economics 9: 811–821. 10.1016/j.forpol.2006.03.009

[2] Liu C, Lobovikov M, Murdiyarso D, Oka H, Youn YC (2005) Paradigm shifts in asian forestry. In: Mery G, Alfaro R, Kanninen M, Lobovikov M, editors. Forests in the global balance—changing paradigms. Vienna: IUFRO World Series. vol. 17. pp. 209–230. Available: http://www.iufro.org/download/file/4247/4451/wfse-achie-artic-13_pdf. Accessed 2014 Dec 14.

[3] Yamada T, Saito H, Fujieda S (2013) Present state of Japanese cedar pollinosis: the national affliction. Journal of Allergy and Clinical Immunology 133: 632–639. 10.1016/j.jaci.2013.11.002 24361081

[4] Imaizumi F, Sidle RC, Kamei R (2008) Effects of forest harvesting on the occurrence of landslides and debris flows in steep terrain of central Japan. Earth Surface Processes and Landforms 33: 827–840. 10.1002/esp.1574

[5] Bellassen V, Luyssaert S (2014) Carbon sequestration: managing forests in uncertain times. Nature 506: 153–155. 10.1038/506153a 24527499

[6] Mac Donncadha M (1997) Japanese forestry and forest harvesting techniques COFORD Available: http://www.coford.ie/media/coford/content/publications/projectreports/japanese.pdf. Accessed 2014 Dec 14.

[7] Grossnickle SC (2012) Why seedlings survive: influence of plant attributes. New Forests 43: 711–738. 10.1007/s11056-012-9336-6

[8] Bonner FT, Karrfalt RP (2008) The woody plant seed manual Washington, DC: USDA Forest Service. 1233 p.

[9] Matsuda K, Miyajima H (1978) Sterile seed formation in sugi *Cryptomeria japonica* D. Don. Journal of the Japanese Forestry Society 60: 1–9. (in Japanese with English summary)

[10] ISTA (1993) International rules for seed testing. Seed Science and Technology 13: 484–487.

[11] Matsuda O, Tanaka A, Fujita T, Iba K (2012) Hyperspectral imaging techniques for rapid identification of *Arabidopsis* mutants with altered leaf pigment status. Plant and Cell Physiology 53: 1154–1170. 10.1093/pcp/pcs043 22470059 PMC3367163

[12] McLafferty FW (2009) Wiley registry of mass spectral data, 9th edition with NIST 2008 [DVD-ROM] Hoboken: John Wiley & Sons.

[13] McGoverin CM, Engelbrecht P, Geladi P, Manley M (2011) Characterisation of non-viable whole barley, wheat and sorghum grains using near-infrared hyperspectral data and chemometrics. Analytical and Bioanalytical Chemistry 401: 2283–2289. 10.1007/s00216-011-5291-x 21842198

[14] Cheng SS, Chang ST (2014) Bioactivity and characterization of exudates from *Cryptomeria japonica* bark. Wood Science and Technology 48: 831–840. 10.1007/s00226-014-0644-1

[15] Jaiswal R, Beuria TK, Mohan R, Mahajan SK, Panda D (2007) Totarol inhibits bacterial cytokinesis by perturbing the assembly dynamics of FtsZ. Biochemistry 46: 4211–4220. 10.1021/bi602573e 17348691

