# Supplementary figures and images for: Determination of Seed Soundness in Conifers Cryptomeria japonica and Chamaecyparis obtusa Using Narrow-Multiband Spectral Imaging in the Short-Wavelength Infrared Range
Source: PLoS One. 2015 Jun 17;10(6):e0128358. doi: 10.1371/journal.pone.0128358 (PMC4470962; doi:10.1371/journal.pone.0128358)

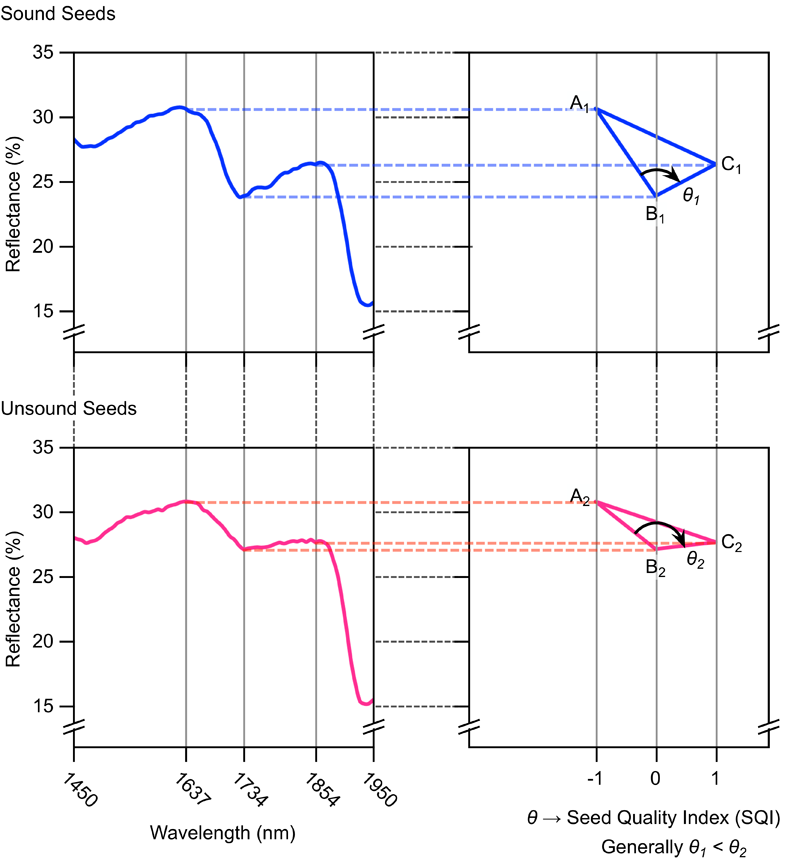

Supplement: S1 Fig — (TIF) [file pone.0128358.s001.tif]

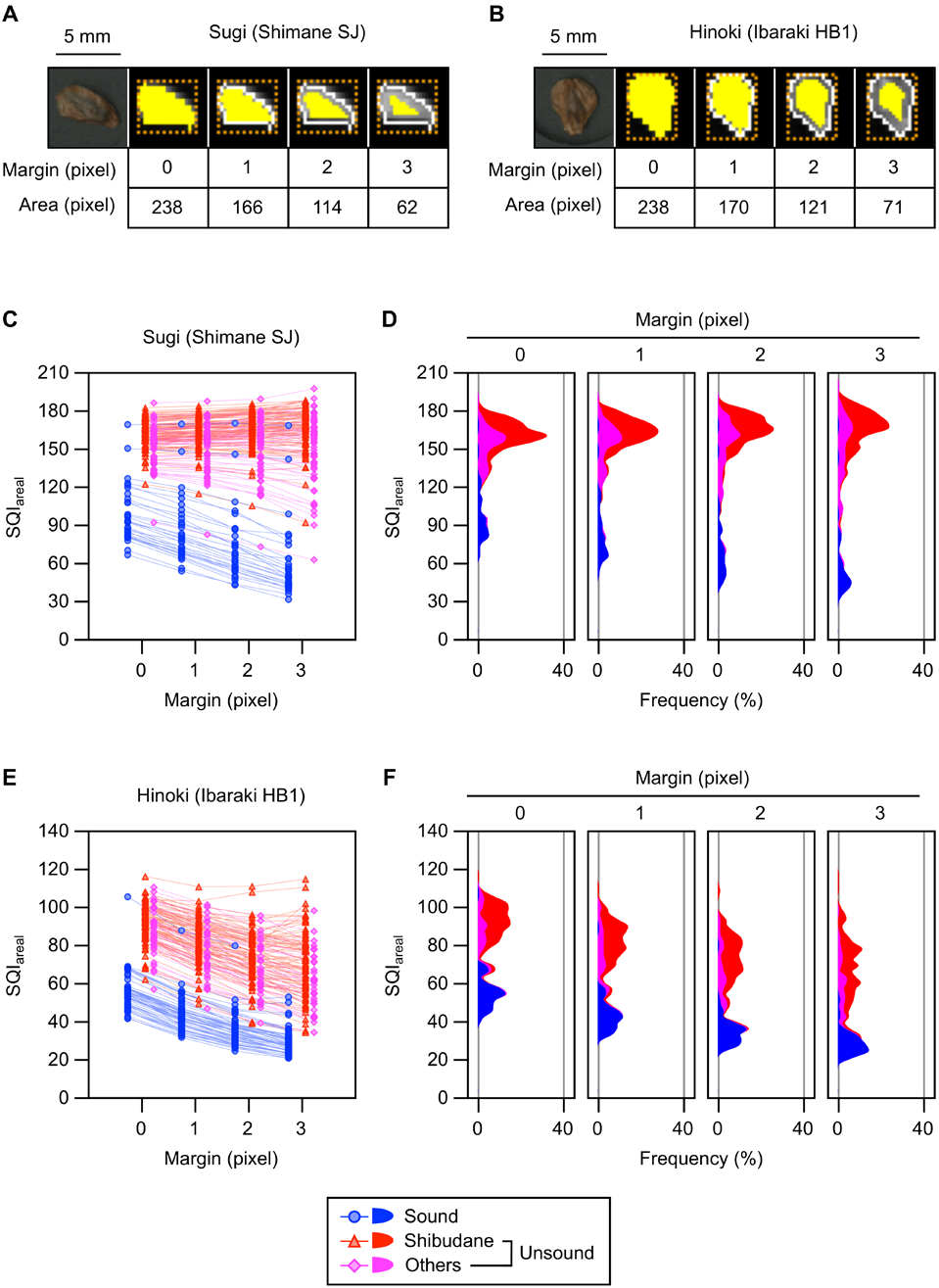

Supplement: S2 Fig — (TIF) [file pone.0128358.s002.tif]
