# Supplementary material for: Determination of Seed Soundness in Conifers Cryptomeria japonica and Chamaecyparis obtusa Using Narrow-Multiband Spectral Imaging in the Short-Wavelength Infrared Range
Source: PLoS One. 2015 Jun 17;10(6):e0128358. doi: 10.1371/journal.pone.0128358 (PMC4470962; doi:10.1371/journal.pone.0128358)
Supplement: S1 Text — (DOCX) [file pone.0128358.s004.docx]

**S1 Text. Legends to S1 File.**

**Software installation**

1. Unzip S1 File (S1File.zip) and run the installation program (setup.exe) extracted in the “S1File\Software” folder.
2. After installation process has been completed, a folder named “Hyperspectral Imaging” will appear on the desktop. For the following operations, open folders “Hyperspectral Imaging\SeedAnalysis (x64)” and “S1File\Data” in separate windows.
3. The software appears in the “Programs and Features” list in the Windows control panel as “Seed Analysis (x64)” and can be uninstalled or repaired from there.

**Operation of SeedSpecAnalyzer**

1. Double-click the shortcut named “SeedSpecAnalyzer” in the “SeedAnalysis (x64)” folder.
2. Drag and drop either hyperspectral image file, “ShimaneSJ.hyp” or “IbarakiHB1.hyp”, available in the “Data” folder onto the SeedSpecAnalyzer application form. If the file has been opened successfully, you will see sugi or hinoki seeds aligned in a 6 × 3 grid format in the image panel.
3. Adjust the diameter of selection cursor in the control panel so that it will not run off the edge of seed area. Then click on one of the seeds in the image panel to capture reference reflectance spectra.
4. Pixels that likely correspond to a seed area will be displayed in blue to red. Adjust the “Filter Setting” track bar in the control panel so that all seed areas will be clearly distinguished from the background. Then proceed to the next step by clicking “►” icon on the top right (or you can return back to the previous steps by clicking “◄” icon).
5. Click the “Start Object Tracking” button in the control panel. You will be prompted with the message, “Are you sure you want to store the areas enclosed in white?” If the contours are extracted precisely, click the “OK” button. Otherwise, click the “Cancel” button and retry the previous steps.
6. Using a chart that appears in the control panel, you can remove stored areas or reassign area numbers with a manual operation. If all 18 seed areas and no other areas are stored properly, input “6” and “3” into the column and row spin buttons, respectively, in the control panel and then click the “Start Renumbering” button. The areas will be renumbered in ascending order from top left to bottom right.
7. Click the “►” icon and a save dialog box appears. Just click the “Save” button and four data files, one text file with an extension of “.hcset” and three bitmap files, will be created in the “Data” folder.
8. The “.hcset” file can be opened in any text editor or in Microsoft® Excel® spreadsheet for viewing and further analysis.

**Operation of SQIViewer**

1. Double-click the shortcut named “SQIViewer” in the “SeedAnalysis (x64)” folder.
2. Drag and drop either hyperspectral image file, “ShimaneSJ.hyp” or “IbarakiHB1.hyp”, available in the “Data” folder onto the SQIViewer application form. If the file has been opened successfully, you will see sugi or hinoki seeds aligned in a 6 × 3 grid format in the picture box.
3. Click the “Seed Quality Index (SQI)” tab. You will see spatial distribution patterns of SQI_pixel_ values within seed areas. Only pixels representing SQI_pixel_ lower than *max*SQI, which is adjustable using a track bar, will be displayed in blue (highest) to red (lowest).
4. Click the “Classified” tab. If both *max*SQI (Step 3) and *min*PBR are adjusted to optimal values using track bar controls, only seeds that are likely to be sound will be highlighted in a saturated color (magenta).
5. Optimal values for *max*SQI and *min*PBR, and the actual positions of sound and unsound seeds are described in “README.txt” file in the “Data” folder.
